# Supplementary material for: Machine Learning Accelerates Crystallization for Structure Determination
Source: Angew Chem Int Ed Engl. 2026 May 4;65(25):e1218503. doi: 10.1002/anie.1218503 (PMC13266958; doi:10.1002/anie.1218503)

# checkCIF/PLATON report

Structure factors have been supplied for datablock(s) lcz058

THIS REPORT IS FOR GUIDANCE ONLY. IF USED AS PART OF A REVIEW PROCEDURE FOR PUBLICATION, IT SHOULD NOT REPLACE THE EXPERTISE OF AN EXPERIENCED CRYSTALLOGRAPHIC REFEREE.

No syntax errors found. CIF dictionary Interpreting this report

# Datablock: lcz058

---

| | | | |
|---|---|---|---|
| Bond precision: | C-C = 0.0106 A | | Wavelength=1.54184 |
| Cell: | a=12.9913(2) | b=22.0768(2) | c=26.2307(2) |
| | alpha=70.203(1) | beta=82.256(1) | gamma=87.704(1) |
| Temperature: | 100 K | | |

| | Calculated | Reported |
|---|---|---|
| Volume | 7013.82(14) | 7013.82(14) |
| Space group | P -1 | P -1 |
| Hall group | -P 1 | -P 1 |
| Moiety formula | C47 H27 Ag3 F18 N6 O2, C31 H15 Ag3 F18 N6 O, 2(C15 H3 Ag3 F18 N | C15 H3 Ag3 F18 N6, C16 H12 O |
| Sum formula | C124 H60 Ag12 F72 N24 O4 | C31 H15 Ag3 F18 N6 O |
| Mr | 4612.40 | 1153.10 |
| Dx,g cm-3 | 2.184 | 2.184 |
| Z | 2 | 8 |
| Mu (mm-1) | 14.566 | 14.566 |
| F000 | 4432.0 | 4432.0 |
| F000’ | 4452.84 | |
| h,k,lmax | 16,27,32 | 16,27,30 |
| Nref | 28198 | 26333 |
| Tmin,Tmax | 0.076,0.233 | 0.426,1.000 |
| Tmin’ | 0.005 | |

Correction method= # Reported T Limits: Tmin=0.426 Tmax=1.000
AbsCorr = MULTI-SCAN

Data completeness= 0.934 Theta(max)= 73.313

R(reflections)= 0.0485( 23002) wR2(reflections)= 0.1288( 26333)

S = 1.065 Npar= 2157

---

The following ALERTS were generated. Each ALERT has the format
**test-name_ALERT_alert-type_alert-level.**
Click on the hyperlinks for more details of the test.

---

### Alert level C

```
PLAT029_ALERT_3_C _diffrn_measured_fraction_theta_full value Low .      0.969 Why?
PLAT041_ALERT_1_C Calc. and Reported SumFormula    Strings  Differ     Please Check
              Calc: C124 H60 Ag12 F72 N24 O4
              Rep.: C31 H15 Ag3 F18 N6 O
PLAT042_ALERT_1_C Calc. and Reported MoietyFormula Strings  Differ     Please Check
              Calc: C47 H27 Ag3 F18 N6 O2, C31 H15 Ag3 F18 N6 O, 2
                   (C15 H3 Ag3 F18 N6), C16 H12 O
              Rep.: C15 H3 Ag3 F18 N6, C16 H12 O
PLAT213_ALERT_2_C Atom F049            has ADP max/min Ratio .....        3.7 prolat
PLAT213_ALERT_2_C Atom F04L            has ADP max/min Ratio .....        3.6 prolat
PLAT214_ALERT_2_C Atom F068      (Anion/Solvent) ADP max/min Ratio        4.2 prolat
PLAT214_ALERT_2_C Atom F06C      (Anion/Solvent) ADP max/min Ratio        4.1 prolat
PLAT214_ALERT_2_C Atom F06K      (Anion/Solvent) ADP max/min Ratio        4.4 prolat
PLAT214_ALERT_2_C Atom F06E      (Anion/Solvent) ADP max/min Ratio        5.0 prolat
PLAT214_ALERT_2_C Atom F04Y      (Anion/Solvent) ADP max/min Ratio        4.4 prolat
PLAT221_ALERT_2_C Solv./Anion  Resd 3  F   Ueq(max)/Ueq(min) Range        5.2 Ratio
PLAT230_ALERT_2_C Hirshfeld Test Diff for    F02V     --C069     .        5.1 s.u.
PLAT234_ALERT_4_C Large Hirshfeld Difference F024     --C06I     .       0.17 Ang.
PLAT234_ALERT_4_C Large Hirshfeld Difference F02C     --C06E     .       0.16 Ang.
PLAT234_ALERT_4_C Large Hirshfeld Difference F0       --C06H     .       0.21 Ang.
PLAT241_ALERT_2_C High   'MainMol' Ueq as Compared to Neighbors of       O02F Check
PLAT250_ALERT_2_C Large U3/U1 Ratio for <U(i,j)> Tensor(Resd    4)        2.1 Note
PLAT342_ALERT_3_C Low Bond Precision on  C-C Bonds ..............     0.0106 Ang.
PLAT790_ALERT_4_C Centre of Gravity not Within Unit-Cell: Resd.  #          1 Note
              C47 H27 Ag3 F18 N6 O2
PLAT906_ALERT_3_C Large K Value in the Analysis of Variance ......      2.047 Check
PLAT910_ALERT_3_C Missing FCF Reflection(s) Below Theta(Min)[Deg]=       3.52 Note
                1  0  0,   0  1  0,   0 -1  1,   0  0  1,   0  1  1,
PLAT911_ALERT_3_C Missing FCF Refl Between Thmin & STh/L=    0.600        759 Report
               -3  5  0,  -1  7  0,   0  0  2,   9-14  3, -12 16  3, -11 18  4,
              -12  6  6, -12  7  6,   5-18 10,  13 -7 14,  13 -6 15,  13 -5 16,
                5 17 16,  11  6 18,   4 19 20,  -1 -6 21,  -4 -3 21,   3-10 22,
                0 -5 22,   3 -5 22,  -3 -3 22,  -4 -1 22,   0  1 22,   1  1 22,
                0  2 22,   0-10 23,   1-10 23,   4-10 23,  -2 -9 23,  -3 -8 23,
                   (  729 More Missing: see the .ckf listing file)
PLAT971_ALERT_2_C Check Calcd Resid. Dens.  1.60Ang From F04L          2.10 eA-3
PLAT971_ALERT_2_C Check Calcd Resid. Dens.  1.20Ang From F068          1.91 eA-3
PLAT971_ALERT_2_C Check Calcd Resid. Dens.  1.01Ang From Ag03          1.61 eA-3
PLAT971_ALERT_2_C Check Calcd Resid. Dens.  1.07Ang From Ag02          1.61 eA-3
```

```
PLAT971_ALERT_2_C Check Calcd Resid. Dens.  1.03Ang From F06C          1.60 eA-3
PLAT971_ALERT_2_C Check Calcd Resid. Dens.  1.03Ang From Ag05          1.57 eA-3
PLAT971_ALERT_2_C Check Calcd Resid. Dens.  0.99Ang From Ag01          1.56 eA-3
PLAT971_ALERT_2_C Check Calcd Resid. Dens.  1.08Ang From Ag0A          1.55 eA-3
PLAT972_ALERT_2_C Check Calcd Resid. Dens.  0.84Ang From Ag08         -1.63 eA-3
PLAT972_ALERT_2_C Check Calcd Resid. Dens.  0.84Ang From Ag04         -1.58 eA-3
PLAT972_ALERT_2_C Check Calcd Resid. Dens.  0.84Ang From Ag0B         -1.56 eA-3
PLAT972_ALERT_2_C Check Calcd Resid. Dens.  0.83Ang From Ag07         -1.56 eA-3
```

---

**Alert level G**

```
PLAT002_ALERT_2_G Number of Distance or Angle Restraints on AtSite          7 Note
PLAT003_ALERT_2_G Number of Uiso or U(i,j)  Restrained non-H-Atoms         10 Report
PLAT045_ALERT_1_G Calculated and Reported Z Differ by a Factor ...      0.250 Check
PLAT083_ALERT_2_G SHELXL Second Parameter in WGHT  Unusually Large      45.18 Why ?
PLAT153_ALERT_1_G The s.u.'s on the Cell Axes   are Equal ..(Note)     0.0002 Ang.
PLAT154_ALERT_1_G The s.u.'s on the Cell Angles are Equal ..(Note)      0.001 Degree
PLAT176_ALERT_4_G The CIF-Embedded .res File Contains SADI Records          3 Report
PLAT186_ALERT_4_G The CIF-Embedded .res File Contains ISOR Records          5 Report
PLAT230_ALERT_2_G Hirshfeld Test Diff for    F06D     --C06H     .       11.1 s.u.
PLAT242_ALERT_2_G Low    'MainMol' Ueq as Compared to Neighbors of       C05E Check
PLAT242_ALERT_2_G Low    'MainMol' Ueq as Compared to Neighbors of       C05F Check
PLAT242_ALERT_2_G Low    'MainMol' Ueq as Compared to Neighbors of       C05M Check
PLAT242_ALERT_2_G Low    'MainMol' Ueq as Compared to Neighbors of       C060 Check
PLAT242_ALERT_2_G Low    'MainMol' Ueq as Compared to Neighbors of       C069 Check
PLAT242_ALERT_2_G Low    'MainMol' Ueq as Compared to Neighbors of       C06I Check
PLAT242_ALERT_2_G Low    'MainMol' Ueq as Compared to Neighbors of       C04H Check
PLAT242_ALERT_2_G Low    'MainMol' Ueq as Compared to Neighbors of       C053 Check
PLAT242_ALERT_2_G Low    'MainMol' Ueq as Compared to Neighbors of       C058 Check
PLAT242_ALERT_2_G Low    'MainMol' Ueq as Compared to Neighbors of       C05J Check
PLAT242_ALERT_2_G Low    'MainMol' Ueq as Compared to Neighbors of       C05N Check
PLAT242_ALERT_2_G Low    'MainMol' Ueq as Compared to Neighbors of       C06A Check
PLAT242_ALERT_2_G Low    'MainMol' Ueq as Compared to Neighbors of       C03N Check
PLAT242_ALERT_2_G Low    'MainMol' Ueq as Compared to Neighbors of       C05X Check
PLAT242_ALERT_2_G Low    'MainMol' Ueq as Compared to Neighbors of       C06B Check
PLAT242_ALERT_2_G Low    'MainMol' Ueq as Compared to Neighbors of       C06E Check
PLAT242_ALERT_2_G Low    'MainMol' Ueq as Compared to Neighbors of       C06J Check
PLAT242_ALERT_2_G Low    'MainMol' Ueq as Compared to Neighbors of       C04B Check
PLAT242_ALERT_2_G Low    'MainMol' Ueq as Compared to Neighbors of       C04U Check
PLAT242_ALERT_2_G Low    'MainMol' Ueq as Compared to Neighbors of       C05S Check
PLAT242_ALERT_2_G Low    'MainMol' Ueq as Compared to Neighbors of       C061 Check
PLAT242_ALERT_2_G Low    'MainMol' Ueq as Compared to Neighbors of       C066 Check
PLAT242_ALERT_2_G Low    'MainMol' Ueq as Compared to Neighbors of       C06F Check
PLAT302_ALERT_4_G Anion/Solvent/Minor-Residue Disorder (Resd    3)         7% Note
PLAT434_ALERT_2_G Short Inter HL..HL Contact F00K     ..F029     .       2.77 Ang.
                                                     x,y,z  =     1_555 Check
PLAT434_ALERT_2_G Short Inter HL..HL Contact F00M     ..F01C     .       2.81 Ang.
                                                   1+x,y,z  =     1_655 Check
PLAT434_ALERT_2_G Short Inter HL..HL Contact F00R     ..F02N     .       2.74 Ang.
                                                  -1+x,y,z  =     1_455 Check
PLAT434_ALERT_2_G Short Inter HL..HL Contact F00Z     ..F04L     .       2.78 Ang.
                                                     x,y,z  =     1_555 Check
PLAT434_ALERT_2_G Short Inter HL..HL Contact F017     ..F06L     .       2.76 Ang.
                                                     x,y,z  =     1_555 Check
PLAT434_ALERT_2_G Short Inter HL..HL Contact F01N     ..F02V     .       2.77 Ang.
                                                   1+x,y,z  =     1_655 Check
PLAT434_ALERT_2_G Short Inter HL..HL Contact F02N     ..F04L     .       2.74 Ang.
```

```
                                                     x,y,z  =      1_555 Check
PLAT434_ALERT_2_G Short Inter HL..HL Contact F05Q     ..F06K     .       2.72 Ang.
                                                   1+x,y,z  =      1_655 Check
PLAT720_ALERT_4_G Number of Unusual/Non-Standard Labels .........        299 Note
              Ag01    Ag02    Ag03    Ag04    Ag05    Ag06    Ag07    Ag08
              Ag09    Ag0A    Ag0B    Ag0C    F00D    F00E    F00F    F00G
              F00H    F00I    F00J    F00K    F00L    F00M    F00N    O00O
              O00P    F00Q    F00R    F00S    F00T    F00U    F00V    N00W
              F00X    N00Y    F00Z    N010    F011    N012    F013    N014
              F015    F016    F017    F018    F019    N01A    F01B    F01C
              F01D    F01E    F01F    F01G    F01H    F01I    N01J    N01K
              N01L    F01M    F01N    N01O    O01P    F01Q    N01R    F01S
              N01T    N01U    N01V    N01W    N01X    N01Y    N01Z    F020
              N021    F022    N023    F024    N025    N026    F027    F028
              F029    F02A    N02B    F02C    F02D    N02E    O02F    F02G
              F02H    F02I    F02J    F02K    C02L    F02M    F02N    F02O
              C02P    H02P    C02Q    F02R    C02S    C02T    C02U    F02V
              C02W    F02X    C02Y    C02Z    C030    H030    F031    C032
              C033    C034    C035    C036    C037    C038    C039    C03A
              C03B    C03C    C03D    C03E    H03E    C03F    H03F    C03G
              H03G    C03H    C03I    H03I    C03J    C03K    H03K    F03L
              C03M    C03N    C03O    H03O    C03P    F03Q    C03R    H03R
              C03S    C03T    C03U    H03U    C03V    H03V    C03W    H03W
              C03X    H03X    C03Y    H03Y    C03Z    C040    C041    C042
              C043    H043    C044    C045    C046    H046    C047    C048
              F049    C04A    H04D    C04B    C04C    H04E    C04D    H04F
              C04E    C04F    C04G    C04H    C04I    C04J    H04J    C04K
              H04K    F04L    C04M    C04N    H04A    H04B    H04C    C04O
              F04P    C04Q    C04R    H04R    C04S    H04S    C04T    H04T
              C04U    C04V    C04W    H04W    C04X    F04Y    C04Z    H04Z
              C050    H050    C051    C052    H052    C053    C054    H054
              F056    C057    H057    C058    C059    H05J    H05K    H05L
              C05A    H05A    C05B    H05B    C05C    H05C    C05D    H05D
              C05E    C05F    C05G    C05H    H05H    C05I    H05I    C05J
              C05K    C05L    H05M    C05M    C05N    C05O    H05O    C05P
              H05P    F05Q    C05R    H05E    H05F    H05G    C05S    C05T
              H05T    C05U    H05U    C05V    H05V    C05W    C05X    C05Y
              H05Y    C05Z    H05Z    C060    C061    C062    C063    H063
              C064    H064    C065    H065    C066    C067    H067    F068
              C069    C06A    C06B    F06C    F06D    C06E    C06F    C06G
              H06A    H06B    H06C    C06H    C06I    C06J    F06K    F06L
              F06M    F06E    F0
PLAT860_ALERT_3_G Number of Least-Squares Restraints ............         63 Note
PLAT883_ALERT_1_G Absent Datum for _atom_sites_solution_primary ..     Please Do !
PLAT912_ALERT_4_G Missing # of FCF Reflections Above STh/L=  0.600        934 Note
PLAT933_ALERT_2_G Number of HKL-OMIT Records in Embedded .res File          2 Note
               -3  5  0,  -1  7  0,
PLAT941_ALERT_3_G Average HKL Measurement Multiplicity ..........        4.5 Low
PLAT952_ALERT_5_G Calculated (ThMax) and CIF-Reported Lmax Differ.          2 Units
PLAT958_ALERT_1_G Calculated (ThMax) and Actual (FCF) Lmax Differ.          2 Units
PLAT969_ALERT_5_G The 'Henn et al.' R-Factor-gap value ..........      5.117 Note
              Predicted wR2: Based on SigI**2  2.52 or SHELX Weight 12.10
PLAT978_ALERT_2_G Number C-C Bonds with Positive Residual Density.          0 Info
```

---

0 **ALERT level A** = Most likely a serious problem - resolve or explain
0 **ALERT level B** = A potentially serious problem, consider carefully

```
 34 ALERT level C = Check. Ensure it is not caused by an omission or oversight
 51 ALERT level G = General information/check it is not something unexpected

  7 ALERT type 1 CIF construction/syntax error, inconsistent or missing data
 60 ALERT type 2 Indicator that the structure model may be wrong or deficient
  7 ALERT type 3 Indicator that the structure quality may be low
  9 ALERT type 4 Improvement, methodology, query or suggestion
  2 ALERT type 5 Informative message, check
```

---

### Validation response form

Please find below a validation response form (VRF) that can be filled in and pasted into your CIF.

```
# start Validation Reply Form
_vrf_PLAT029_lcz058
;
PROBLEM: _diffrn_measured_fraction_theta_full value Low .      0.969 Why?
RESPONSE: ...
;
_vrf_PLAT041_lcz058
;
PROBLEM: Calc. and Reported SumFormula    Strings  Differ     Please Check
RESPONSE: ...
;
_vrf_PLAT042_lcz058
;
PROBLEM: Calc. and Reported MoietyFormula Strings  Differ     Please Check
RESPONSE: ...
;
_vrf_PLAT213_lcz058
;
PROBLEM: Atom F049            has ADP max/min Ratio .....        3.7 prolat
RESPONSE: ...
;
_vrf_PLAT214_lcz058
;
PROBLEM: Atom F068      (Anion/Solvent) ADP max/min Ratio        4.2 prolat
RESPONSE: ...
;
_vrf_PLAT221_lcz058
;
PROBLEM: Solv./Anion  Resd 3  F   Ueq(max)/Ueq(min) Range        5.2 Ratio
RESPONSE: ...
;
_vrf_PLAT230_lcz058
;
PROBLEM: Hirshfeld Test Diff for    F02V     --C069     .        5.1 s.u.
RESPONSE: ...
;
_vrf_PLAT234_lcz058
;
PROBLEM: Large Hirshfeld Difference F024     --C06I     .       0.17 Ang.
RESPONSE: ...
;
_vrf_PLAT241_lcz058
```

```
;
PROBLEM: High   'MainMol' Ueq as Compared to Neighbors of       O02F Check
RESPONSE: ...
;
_vrf_PLAT250_lcz058
;
PROBLEM: Large U3/U1 Ratio for <U(i,j)> Tensor(Resd    4)        2.1 Note
RESPONSE: ...
;
_vrf_PLAT342_lcz058
;
PROBLEM: Low Bond Precision on  C-C Bonds ...............     0.0106 Ang.
RESPONSE: ...
;
_vrf_PLAT790_lcz058
;
PROBLEM: Centre of Gravity not Within Unit-Cell: Resd.  #          1 Note
RESPONSE: ...
;
_vrf_PLAT906_lcz058
;
PROBLEM: Large K Value in the Analysis of Variance ......      2.047 Check
RESPONSE: ...
;
_vrf_PLAT910_lcz058
;
PROBLEM: Missing FCF Reflection(s) Below Theta(Min)[Deg]=       3.52 Note
RESPONSE: ...
;
_vrf_PLAT911_lcz058
;
PROBLEM: Missing FCF Refl Between Thmin & STh/L=    0.600        759 Report
RESPONSE: ...
;
_vrf_PLAT971_lcz058
;
PROBLEM: Check Calcd Resid. Dens.  1.60Ang From F04L            2.10 eA-3
RESPONSE: ...
;
_vrf_PLAT972_lcz058
;
PROBLEM: Check Calcd Resid. Dens.  0.84Ang From Ag08           -1.63 eA-3
RESPONSE: ...
;
# end Validation Reply Form
```

---

It is advisable to attempt to resolve as many as possible of the alerts in all categories. Often the minor alerts point to easily fixed oversights, errors and omissions in your CIF or refinement strategy, so attention to these fine details can be worthwhile. It is up to the individual to critically assess their own results and, if necessary, seek expert advice.

---

**PLATON version of 17/09/2025; check.def file version of 17/09/2025**

---

# duplicate check

## No duplication found

---

**Datablock lcz058** - ellipsoid plot

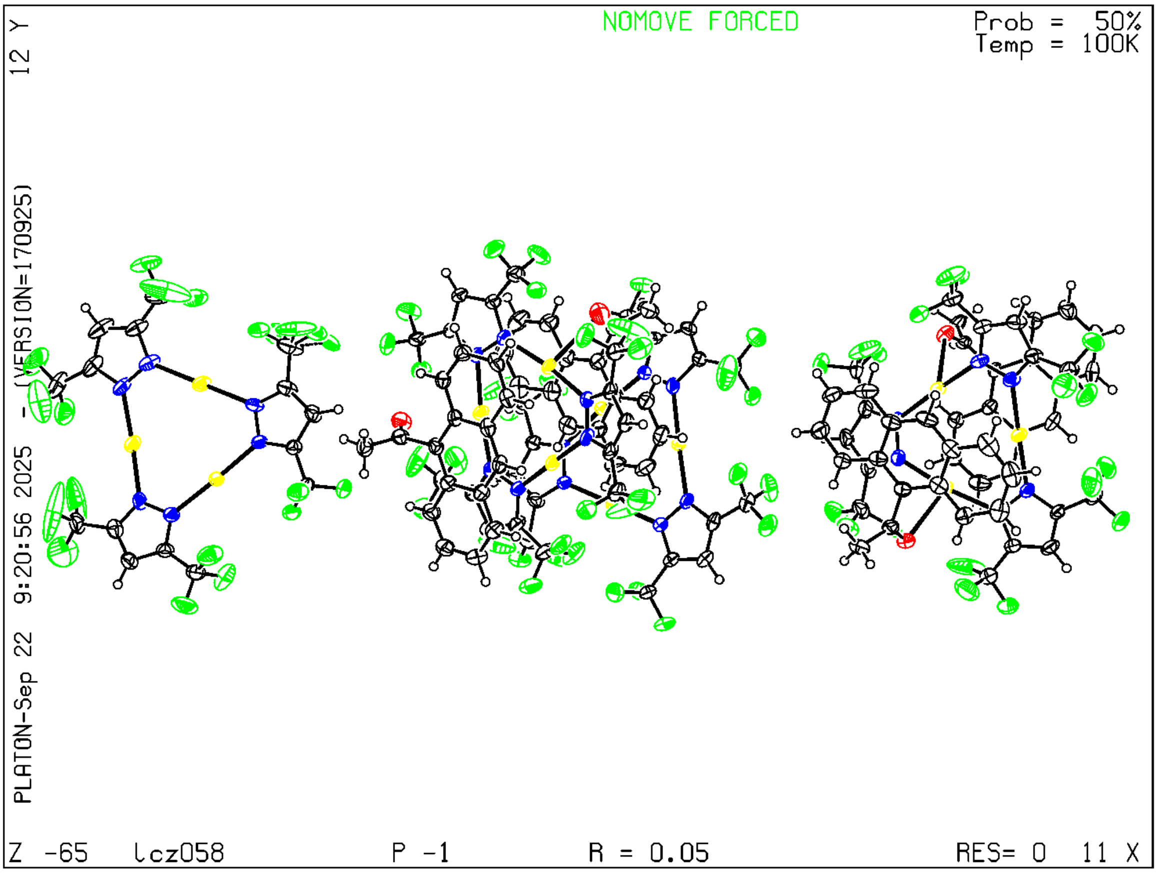

Supplement: Supplementary file 2 — Supporting File 2: anie72462‐sup‐0002‐DataFile.zip. [file ANIE-65-e1218503-s002.zip › supplementary_materials_crystallographic_data submit.zip!/cif_and_checkcif(1-114)/Ag3Pz3_58/Ag3Pz3_58_checkcif.pdf]
